# The influence of local pain on balance control in patients with chronic ankle instability

**DOI:** 10.1186/s12891-022-05656-4

**Published:** 2022-07-22

**Authors:** Yungu Chen, Shengxuan Cao, Lewen Qian, Wenming Chen, Chen Wang, Xin Ma, Xu Wang, Jiazhang Huang

**Affiliations:** 1 Department of Orthopedics, Huashan Hospital, Fudan University, 12 Middle Wulumuqi Road, Jingan District, Shanghai, China; 2 Institute of Biomedical Engineering, Academy for Engineering & Technology, Fudan University, 220 Handan Road, Yangpu District, Shanghai, China

**Keywords:** Chronic ankle instability, Pain, Balance control, Star excursion balance test, Single-leg stance, Center of pressure, Electromyography

## Abstract

**Background:**

Local pain around the ankle joint is a common symptom in patients with chronic ankle instability (CAI). However, whether the local pain would impose any influence on the balance control performance of CAI patients is still unknown.

**Methods:**

A total of twenty-six subjects were recruited and divided into the following two groups: pain-free CAI (group A) and pain-present CAI (group B). Subjects in both groups received two independent tests: the star excursion balance test and the single-leg stance test, in order to reflect their balance control ability more accurately.

**Results:**

Compared with group A, the group B showed significantly more episodes of the history of sprains, decreased ankle maximum plantarflexion angle, and lower Cumberland scores (all *p* < 0.05). In the star excursion balance test, group B demonstrated a significantly reduced anterior reach distance than group A (*p* < 0.05). During the single leg stance test, group B showed a significant increase in the magnitude of electromyographic signals both in peroneus longus and soleus muscles than group A (each *p* < 0.05). Additionally, group B had a significantly more anterolaterally positioned plantar center of pressure than group A (*p* < 0.05).

**Conclusion:**

CAI patients with local pain around the ankle joint had more episodes of sprains and lower functional scores when compared to those without pain. The balance control performance was also worse in the pain-present CAI patients than those without pain.

## Background

Lateral ankle sprains are the most common musculoskeletal injuries in both physically active populations and general populations [1], thereby posing a major clinical challenge and healthcare burden. The typical injury mechanism is a sudden force of inversion and internal rotation that injure the ankle joint complex [1]. Nearly 20% of patients with lateral ankle sprains develop long-term symptoms including pain, giving way, sense of instability and recurrent sprains, which contribute to chronic ankle instability (CAI) [2].

It has already been well documented that CAI patients were associated with impaired balance control, due to proprioception and neuromuscular deficits [3–5]. The impairment of balance control has been identified utilizing a variety of approaches, including the star excursion balance test (SEBT) [6] and the single leg stance test [7]. The SEBT has been used to compare balance ability among different sports and identify individuals who have CAI [8]. Hubbard et al. [9] reported that the anterior and posteromedial reach directions identified persons with CAI. Deficits in balance control are also reflected by single-leg stance. Ross et al. [10] documented increased displacement of the body center of pressure (CoP) when individuals with CAI balanced in a single-leg stance. Results of these measures provide an insight into the balance-control strategy used in CAI patients.

Pain is reported to occur in 58% of patients with CAI when they participate in different levels of activity [11]. However, its impact on the ankle function and balance performance of CAI patients is still unknown. A recent study has reported that for individuals with CAI, ankle pain is associated with perceived ankle instability [12] and worse ankle function [13]. However, limited evidence has shown the influence of the ankle local pain on the balance control performance of patients with CAI.

This study was designed to investigate the influence of local pain around ankle joint on balance control of the CAI patients through the SEBT and the single-leg stance test. We hypothesized that CAI patients with local pain would have worse balance control performance than pain-free CAI patients and healthy individuals.

## Methods

### Participants

A total of twenty-six subjects with self-reported CAI were recruited in this study from December 2021 to March 2022. Twelve subjects were assigned to the pain-free CAI group (group A), and fourteen subjects were assigned to the pain-present CAI group (group B). This study was approved by our institutional review board. All subjects provided written informed consent. No conflicts of interest were existing and this was disclosed to all recruited subjects.

Sample size was calculated by the formula: sample size = (Z_1-α/2_)^2^(SD)^2^ /d^2^ [14]. Z_1-α/2_ is standard normal variate, which equals 1.96 at 5% type I error; SD is standard deviation of variable taken from previous study; d is absolute error or precision decided by researchers. SD was 1.7 according to a previous study that recorded Visual Analog Scale of pain of CAI patients [15], and d was set as 1. Thereafter, the sample size was calculated to be at least 11 ankles in each group.

The inclusion and exclusion criteria were designed in accordance with previous literature [16, 17]. Participants were defined as CAI patients if all the following criteria were met: (1) age within 18 to 50 years; (2) at least one episode of significant ankle sprain sustained no less than 12 months prior to the recruitment; (3) at least one interrupted day of desired physical activity; (4) existing residual symptoms including recurrent ankle sprains, and/or at least two episodes of sprains and/or perceived ankle instability in the previous 6 months; and (5) Cumberland ankle instability tool (CAIT) [18] scores lower than 24. CAIT is a nine-item questionnaire that measures severity of functional ankle instability with excellent validity and reliability [18]. The level of ankle instability is reported in different activities, including running, walking, hopping, and descending stairs. The total CAIT score ranges from 0 to 30, in which 30 represents normal stability. The total Exclusion criteria were as follows: (1) a history of fracture requiring realignment or musculoskeletal surgery in either lower extremity; (2) osteoarthritis in either lower extremity, head trauma, inner ear disease, muscular dystrophy, or other conditions that could affect balance control; and (3) a history of acute injury to the lower extremity within 3 months before the enrollment.

The local pain around the ankle joint was assessed by the 11-point Numeric Rating Scale (NRS-11) [19]. The NRS-11 is a segmented numeric version of the Visual Analog Scale (VAS) in which a respondent selects a whole number (0–10 integers) that best reflects the intensity of their pain [20]. Previous literature related to CAI that measured pain intensity on a numeric rating scale used a score > 0 to define the presence of pain [21–23]. Therefore, the presence of pain was determined if subjects rated their pain as no less than 1. Next, the location of ankle pain was determined by asking subjects to point to the area where they experienced the maximum pain. These evaluations were performed by two independent orthopedic surgeons. For participants with CAI to be categorized as pain-present CAI patients, they had to be considered as having pain by both clinicians.

### Experimental protocol

Senior orthopedic surgeons examined the subjects and documented their age, sex, height, weight, and ankle sprain history. The scores of CAIT and the NRS-11 for pain of each subject were also documented. A physical examination of each subject’s ankle was performed to measure the active range of motion (ROM) in a non-weight bearing position.

The dynamic balance control was assessed by the SEBT [6]. The subjects’ height, weight, and lower limb length were measured. Performance on the SEBT requires a subject to move from a position of bilateral stance to a position of single-leg stance whereby the non-test limb was used to reach maximally, making a light touch along one of three designated lines on the ground, and then return to a position of bilateral stance [24]. One line was oriented anteriorly to the apex and the other two aligned at 135º to this in the posterolateral and posteromedial directions. For each subject, the side with CAI was tested barefoot. Three reach distances in each direction were recorded and normalized to lower limb length by calculating the maximized reach distance (%MAXD) using the formula (Reach distance/limb length) × 100 = %MAXD [25]. The average of the three reach trials in each direction was calculated.

To assess the static balance control, subjects were instructed to perform a single-leg stance on the force plate for 20 s, using the same tested leg as in SEBT. For each test, the CoP sway parameters and raw electromyographic (EMG) data of the last 10 s were recorded. The single-leg stance was repeated for three times and the average results were calculated for further analysis. The validity and reliability of the force plate [26] and EMG device [27] used in this study were testified by previous studies.

The raw EMG data of tibialis anterior (TA), peroneus longus (PL), soleus (SOL), and medial gastrocnemius (MG) were collected by using the OpenSignals surface myoelectric device (Biosignalsplux, Plux, Portugal) at a sampling rate of 1000 Hz. The raw EMG signals were subsequently bandpass filtered (frequency ranging from 10 to 500 Hz) and then passed through the 48–52 Hz notch to reduce power frequency interference [28]. The intersection of wires of each channel was avoided to prevent cross-talk. The mean EMG root mean square (RMS_EMG_) values of each channel were calculated by customized programs in MatLab (The Mathworks, Natick, MA, USA). The RMS_EMG_represents the average level of EMG signals; thus it reflects the activation characteristics of the tasking muscles [29].

**Fig. 1:**
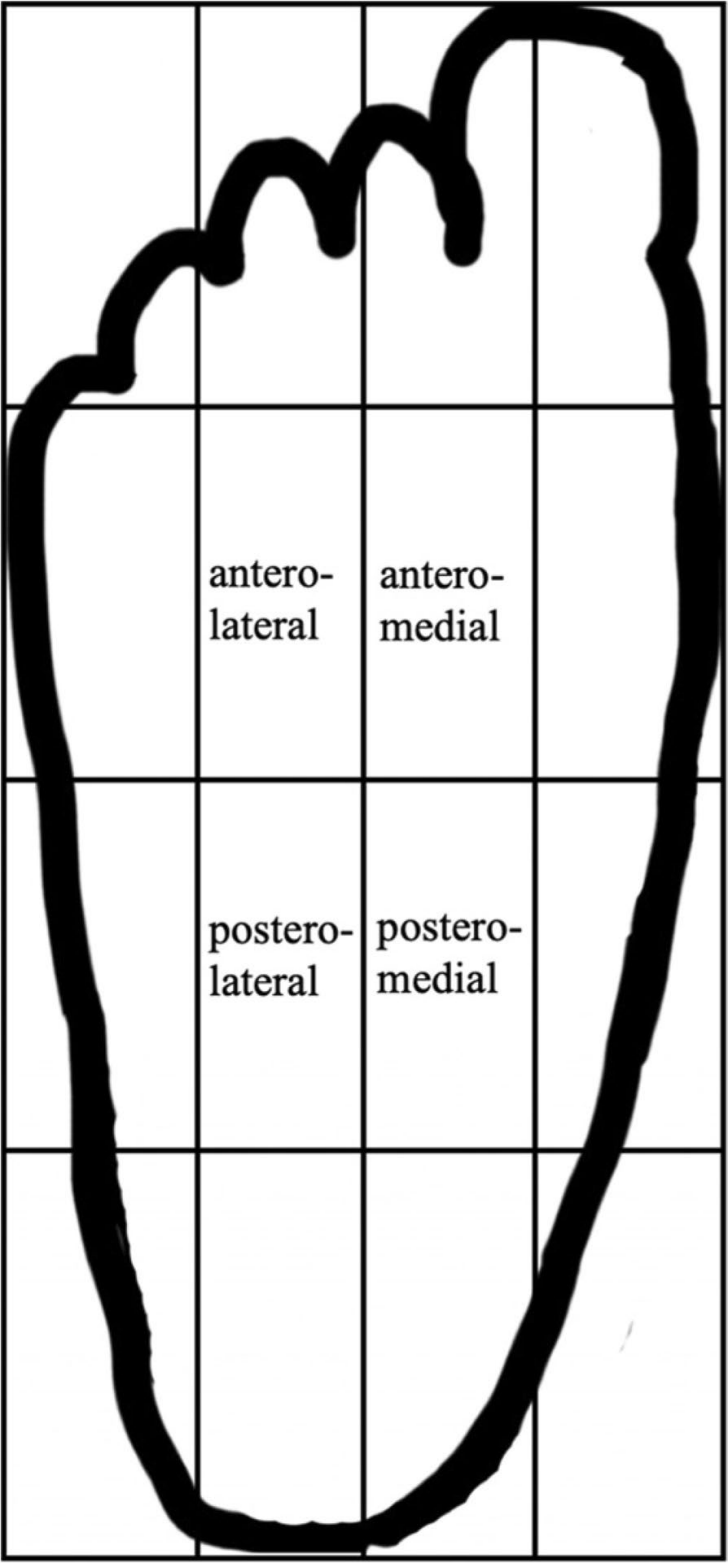
Distribution of plantar CoP data points. If plantar surface of each participant’s foot was divided into 16 equal regions, it was demonstrated that all plantar CoP data points were in the 4 innermost regions during single-leg stance. Abbreviations: CoP: center of pressure

The CoP sway parameters included anteroposterior (AP) amplitude, standard deviation (SD) of AP amplitude, mediolateral (ML) amplitude, SD of ML amplitude, mean velocity of CoP, SD of mean velocity, CoP path and CoP area [28, 30, 31]. CoP data were recorded as coordinates in the AP and ML directions with a sampling frequency of 1000 Hz by the force plate. CoP area was calculated by fitting a 95% confidence ellipse to the collective CoP data [28]. In addition, the plantar surface of each participant’s foot was divided into 16 equal regions. (Fig. 1) Previous literature demonstrated that all CoP data points were located in the four innermost sections during a single-leg stance [32, 33]. The location of each CoP data point was recorded relative to the four innermost sections. The frequency of CoP data points in each section was counted for further analysis.

### Statistical analysis

The statistical analysis was performed using IBM SPSS Statistics 24.0. Q-Q map and Kolmogorov–Smirnov tests were used to assess the normality of CoP and EMG data distribution; both CoP and EMG data passed the normality test. Differences in clinical and demographic parameters between group A and B were evaluated by two-tailed independent t-test except sex, which was a categorical variable and was analyzed by Pearson Chi-square test. All biomechanical parameters were continuous variables and were analyzed using two-tailed independent t-test. The level of significance was set a priori at *p* < 0.05.

## Results

A comparison of the participants’ demographic characteristics between group A (pain-free CAI) and group B (pain-present CAI) is shown in Table 1. Total episodes of the history of sprains in group B were significantly higher than those in group A (*p* < 0.05). In terms of ROM of ankle joints, it was found that group B had significantly lower maximum plantarflexion angle than group A (*p* < 0.05). Moreover, group B had significantly lower CAIT scores than group A (*p* < 0.05). The occurrence of pain in subjects with CAI was 53.8% (14/26). With respect to the local pain around the ankle joint in group B, the mean NRS-11 score was 4.29. The highest pain percentage was reported when running on uneven surfaces (35.7%). Anterior aspect of the lateral malleolus was the most frequently reported site of pain (35.7%), whereas only 1 individual reported deep pain at the lateral joint area (7.1%) (Table 2).

**Table 1:** Demographic and clinical characteristics of subjects in the two groups

Demographic and clinical parameters	Group A (*n* = 12)	Group B (*n* = 14)	*P* value
Female/male^a^	5/7	8/6	0.431
Age (years)	27.33 (4.55)	27.86 (4.04)	0.686
Weight (kg)	72.10 (15.02)	70.32 (15.84)	0.716
Height (cm)	178.28 (8.67)	174.46 (7.80)	0.142
Body mass index (kg/m^2^)	22.44 (3.38)	22.99 (4.58)	0.669
Episodes of the history of sprains	3.00 (1.19)	5.50 (3.19)	**0.001**
Time since last sprain (months)	11.50 (5.99)	12.50 (7.61)	0.636
Maximum dorsiflexion angle (°)	20.50 (6.74)	18.38 (11.41)	0.456
Maximum plantarflexion angle (°)	55.00 (7.28)	48.75 (12.18)	**0.045**
CAIT scores	22.50 (1.86)	11.50 (6.60)	** < 0.001**
NRS-11 scores	0 (0)	4.29 (1.88)	** < 0.001**

^a^ Statistical difference was calculated by Pearson Chi-square test

Group A: pain-free CAI; group B: pain-present CAI

Parameters are presented as mean (SD) except sex

*Abbreviations: CAI* Chronic ankle instability, *CAIT* Cumberland ankle instability tool, *NRS-11* Numeric Rating Scale

**Table 2:** Reports of pain in the two groups. The level of pain was determined by CAIT question 1

	Group A (*n* = 12)		Group B (*n* = 14)	
	n	%	n	%
CAIT question 1: I have pain in my ankle				
Never	12	100	0	0
During sport			2	14.3
Running on uneven surfaces			5	35.7
Running on level surfaces			3	21.4
Walking on uneven surfaces			3	21.4
Walking on level surfaces			1	7.2
Sites of local pain	N/A			
Anterolateral corner			4	28.6
Tip of the lateral malleolus			2	14.3
Anterior aspect of the lateral malleolus			5	35.7
Posterior aspect of the lateral malleolus			2	14.3
Deep pain at the lateral joint area			1	7.1

Group A: pain-free CAI; group B: pain-present CAI

*Abbreviations: CAI* Chronic ankle instability, *CAIT* Cumberland ankle instability tool, *N/A* Not applicable

The results of SEBT are presented in Table 3. Subjects in group B had a significantly lower anterior %MAXD than those in group A (*p* < 0.05). However, no significant differences were found in the SEBT reach distances in posterolateral and posteromedial directions between the two groups.

**Table 3:** Results of star excursion balance test (SEBT)^a^ in the two groups

SEBT parameters	Group A (*n* = 12)	Group B (*n* = 14)	*P* value
Anterior %MAXD	77.0 (9.2)	64.5 (8.9)	** < 0.001**
Posterolateral %MAXD	73.8 (20.2)	70.5 (7.6)	0.513
Posteromedial %MAXD	80.2 (16.2)	77.1 (7.1)	0.465

*Abbreviations: CAI* Chronic ankle instability, *SEBT* Star Excursion Balance Test, *%MAXD* Maximized reach distance

Group A: pain-free CAI; group B: pain-present CAI

Parameters are presented as mean (SD)

^a^ SEBT results are normalized by length of the lower limb: (Reach distance/limb length) × 100 = %MAXD

**Fig. 2:**
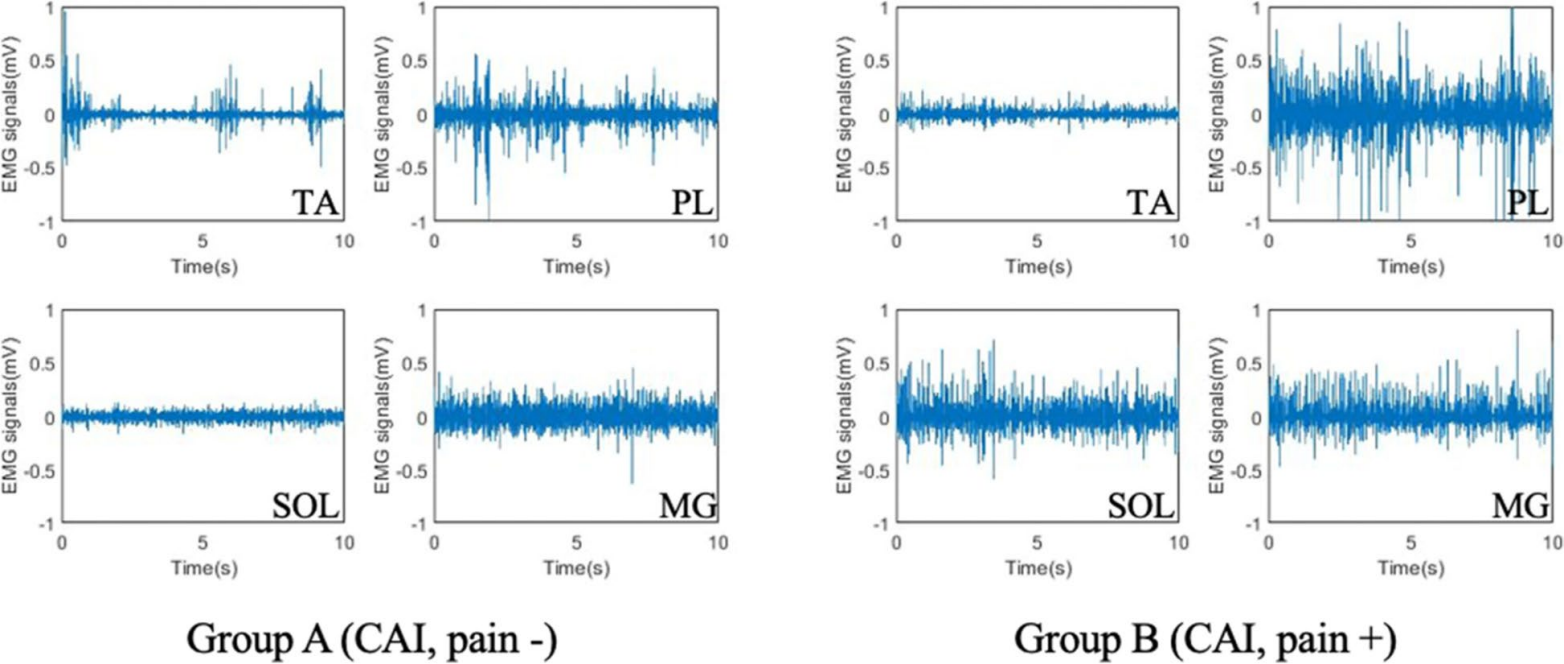
Representatives of EMG of the groups A and B during a single-leg stance. Abbreviations: TA: tibialis anterior; PL: peroneus longus; SOL: soleus; MG: medial gastrocnemius; CAI: chronic ankle instability

Compared to group A, the mean RMS_EMG_ value of PL was significantly larger in group B (*p* < 0.05). Further, significant differences were also found in the mean RMS_EMG_ values of SOL between group A and B (*p* < 0.05, Table 4). Representative images of EMG of the three groups are depicted in Fig. 2.

**Table 4:** The root mean square (RMS_EMG_) values of tibialis anterior, peroneus longus, soleus and medial gastrocnemius, muscles of the two groups

RMS_EMG_	Group A (*n* = 12)	Group B (*n* = 14)	*P* value
Tibialis anterior (mV)	0.050 (0.015)	0.069 (0.048)	0.162
Peroneus longus (mV)	0.122 (0.057)	0.181 (0.078)	**0.039**
Soleus (mV)	0.056 (0.014)	0.085 (0.037)	**0.018**
Medial gastrocnemius (mV)	0.079 (0.036)	0.089 (0.031)	0.425

Group A: pain-free CAI; group B: pain-present CAI

Parameters are presented as mean (SD)

*Abbreviations: CAI* Chronic ankle instability, *RMS*_*EMG*_ mean root mean square of electromyography

The results of CoP parameters of the two groups are summarized in Table 5. In terms of sway parameters, no significant differences were found between group A and B. It was found that all CoP data points were located in the 4 innermost sections. There were notable differences in the CoP data point distribution in the anterolateral section between the two groups, and group B had a higher frequency than group A (*p* < 0.05).

**Table 5:** The CoP parameters of the two groups

CoP parameters	Group A (*n* = 12)	Group B (*n* = 14)	*P* value
Sway parameters			
AP amplitude (cm)	1.23 (0.35)	1.51 (0.53)	0.117
SD of AP amplitude (cm)	0.866 (0.278)	1.135 (0.456)	0.080
ML amplitude (cm)	0.25 (0.04)	0.29 (0.10)	0.183
SD of ML amplitude (cm)	0.178 (0.035)	0.191 (0.047)	0.469
Mean velocity (cm/s)	12.25 (2.48)	12.43 (1.88)	0.836
SD of mean velocity (cm/s)	7.363 (1.897)	9.141 (2.879)	0.285
CoP path (cm)	122.61 (24.70)	124.30 (18.82)	0.845
CoP area (cm^2^)	9.33 (3.96)	13.91 (7.78)	0.068
Section of CoP location (%)			
Anteromedial	18.2 (8.7)	15.8 (6.5)	0.428
Anterolateral	31.1 (7.6)	40.1 (9.6)	**0.016**
Posterolateral	29.6 (8.1)	28.7 (6.4)	0.766
Posteromedial	21.3 (5.0)	15.5 (10.3)	0.075

Group A: pain-free CAI; group B: pain-present CAI

Parameters are presented as mean (SD)

*Abbreviations: CAI* Chronic ankle instability, *CoP* Center of pressure, *AP* Anteroposterior, *SD* Standard deviation, *ML* Mediolateral

## Discussion

In our study, this first finding was that pain-present CAI patients exhibited decreased SEBT anterior reach distance. This may demonstrate the hypothesis that pain-present CAI patients had worse dynamic balance control. The positive association between pain and joint instability has been recognized in other body areas such as shoulder [34]. This was explained by the central alterations when people felt shoulder pain without any nociceptive inputs [34]. In the ankle joint, CAI was found to be associated with negative alterations in supraspinal aspects of motor control [35]. The impact of pain in the ankle joint may be a factor that results in impaired dynamic balance control and recurrent episodes of sprains.

EMG and CoP data were used to quantitively measure the static balance control. Another finding of our study was that pain-present CAI patients had higher levels of muscle activities of PL and SOL during a single-leg stance. It has been found that chronic pain is associated with neural network sensitization that promotes pain and altered nociceptive pathways [36]. Further, pain from the nociceptors at the ankle may worsen the deficits in kinesthesia, joint position sense and force sense of the ankle joints with CAI. Activations of PL and SOL increased muscle-spindle sensitivity [37], which provided the pain-present CAI patients with a better ability to compensate for sensorimotor deficits of the ankle. However, such pattern of muscle activation might also lead to a more rigid and ‘frozen’ ankle joint, which would lose the ability of postural adjustments especially during an episode of perturbation.

With respect to CoP, the CoP sway parameters were not significantly different between group A and B (pain-free vs. pain-present CAI), which might indicate that both groups had impaired static balance control at a close level. Furthermore, it was noted that pain-present CAI patients had a more anterolaterally positioned CoP. Pope et al. [33] and Mettler et al. [32] have already demonstrated that individuals with CAI had more anterolaterally shifted CoP than healthy controls. The authors attributed this finding to a more supinated ankle which maintained rigidity during a single-limb stance. It was proposed that CAI patients sought to constrain the motion at the ankle to accomplish this task [33]. A supinated ankle in a single-leg stance may help stabilize the talocrural and subtalar joints and be potentially beneficial for the prevention of recurrent sprains [33]. However, an anteriorly situated CoP may undermine a person’s ability to maintain balance in the event of a perturbation due to the inadequacy of time and space needed for postural adjustments in this case. In addition, it has been observed that in clinical practice, passive inversion and plantar flexion of ankle joints are more likely to provoke pain when performing a physical examination in patients with CAI [38, 39]. Kwon et al. [39] suggested that pain in a plantar-flexed position might be correlated with a complete tear of the anterior talofibular ligament. CAI patients with pain might have sustained more severe injuries to lateral ankles, forcing them to choose a more dorsiflexed and supinated position of the ankle in a single-limb stance, which consequently resulted in a more anterolaterally placed CoP. This in turn increased the risk of recurrent sprains in the future. In our study, CAI patients with pain indeed had significantly more episodes of the history of sprains than those without pain (Table 1).

The highlight of our study was that this was the first time that the influence of local pain on the balance performance of ankles with CAI was investigated with biomechanical measures. Pain had been a neglected factor in previous studies related to CAI. Our work demonstrated that the presence of ankle pain in CAI patients resulted in worse balance control. However, we recognized that one limitation of our study was the relatively small sample size. This may affect the generalization of the results. Our work was a preliminary study that investigated the influence of local pain on balance control of CAI patients. Future large-sample studies are required to validate the findings of this study.

The SEBT has been described as a reliable measure of dynamic balance control. Decreased reach distances of the SEBT and altered proximal joint neuromuscular control has been observed in individuals with CAI [40]. Most CAI literature utilizing the SEBT did not use pain as a primary outcome. Therefore, the influence of local pain around the ankle on balance control is still unclear. Simsek et al. [41] included twenty-six participants with unilateral CAI and treated them with fibular taping technique. They found that distal fibular taping technique decreased pain intensity and increased SEBT reach distances. Further, a statistically significant negative correlation was documented between the intensity of pain and the SEBT anterior reach distance [41]. The authors concluded that dynamic balance in individuals with CAI was improved by a reduction in pain intensity. However, Terada et al. [42] found no correlations between pain VAS and the SEBT in all three directions. The authors suggested that altered function on the SEBT due to CAI did not necessarily associate with self-reported pain in the ankle. However, it should be noted that VAS scores of pain in their study were low (mean, 2.70), thus creating a floor effect, which may not be able to demonstrate correlations. Schaefer et al. [43] found concomitant improvements in both pain and the SEBT performance after treating CAI patients with soft-tissue mobilization technique. But they did not research into the correlations between pain and the SEBT.

Static balance can be assessed by the single-leg stance test [44]. Literature that assesses the influence of pain on balance control in CAI patients using the single-leg stance test is still limited. Thompson et al. [45] recruited individuals with CAI, copers and healthy individuals to examine their spinal reflexes of the lower limb muscles during static single-leg stance, and it was found that individuals with CAI presented with an inability to suppress soleus spinal reflexes, which was not present in copers and healthy individuals. The authors also found that the presence of pain was partially correlated with this disinhibition. They concluded that pain explained a significant proportion of changes in spinal-level sensorimotor control, which increased the risk of recurrent sprains [45].

Pain is one of the major characteristics of chronic musculoskeletal conditions [46]. Nevertheless, pain has not been a major focus in the CAI literature or a diagnostic criterion of CAI. A recent systematic review revealed that 58% of CAI patients reported pain, and more than 66% of these individuals had persistent pain longer than 6 months [11]. Konradsen et al. [47] reported that more than 70% of CAI patients had pain at the lateral part of the ankle joint complex. Although the pain associated with CAI was mostly described as “mild and intermittent” [48, 49], it has been demonstrated that pain was associated with lower ankle function [12]. Recent pieces of literature focusing on CAI and pain tried to explain this by kinesiophobia [50]. Patients with CAI were found to have injury-related fear that prevented them from rehabilitation training and returning to sports, leading to disuse of function, which further limited the recovery of the functional activity of ankles [51]. Suttmiller et al. [23] recruited 126 CAI patients and evaluated the results of multiple pain and ankle function questionnaires. It was concluded that pain was strongly associated with worse ankle function, and pain might be a predictive factor of decline in the function of ankle joints.

Pain is reported to occur in 58% of patients with CAI [11]. The etiology that some CAI patients experience more pain is probably multifactorial. Shin et al. [52] reported that CAI patients who had therapist-guided exercise showed significantly lower level of pain than those who were prescribed self-management regimen. This indicates that insufficient exercise after sprains tends to result in severer pain. Komenda et al. [53] recruited 54 CAI patients who all had pain and treated them with arthroscopic surgery. It was reported that 93% of ankles had intra-articular abnormalities including synovitis, loose bodies, ossicles, chondromalacia, synovitis, osteochondral lesions of the talus and osteophytes. Intra-articular abnormalities may be a factor that leads to long-term chronic pain after an initial sprain in CAI patients.

Our work proved that pain was a significant factor that contributed to the deficits in both static and dynamic balance control of the ankle joint in people with CAI. A reduction in pain may improve the balance control and prevent recurrent sprains in people with CAI. Future studies are needed to investigate the effect of pain reduction therapies on the improvement of CAI.

## Conclusion

In this study, it was found that pain-present CAI patients had more episodes of sprains and lower functional scores when compared to those without pain. Pain-present CAI patients also had worse dynamic and static balance control than those without pain. In the presence of ankle pain, CAI patients had a more anterolaterally positioned CoP on the plantar surface, and a different activation pattern of lower leg muscles, which might have contributed to worse balance control.

## Data Availability

The datasets generated and/or analysed during the current study are not publicly available due to the patient privacy policy of our institution but are available from the corresponding author on reasonable request.

## Abbreviations

CAI: Chronic ankle instability
SEBT: Star excursion balance test
CoP: Center of pressure
CAIT: Cumberland ankle instability tool
NRS-11: Numeric rating scale
VAS: Visual Analog Scale
ROM: Range of motion
%MAXD: Maximized reach distance
EMG: Electromyography
TA: Tibialis anterior
PL: Peroneus longus
SOL: Soleus
MG: Medial gastrocnemius
RMS_EMG_: Mean root mean square of electromyography
AP: Anteroposterior
SD: Standard deviation
ML: Mediolateral

## References

[1] Gribble PA, Bleakley CM, Caulfield BM (2016). Evidence review for the 2016 International Ankle Consortium consensus statement on the prevalence, impact and long-term consequences of lateral ankle sprains. Br J Sports Med.

[2] Mailuhu AKE, Oei EHG, van Putte-Katier N (2018). Clinical and radiological predictors for persistent complaints five years after a lateral ankle sprain: a long-term follow-up study in primary care. J Sci Med Sport.

[3] Hertel J (2002). Functional anatomy, pathomechanics, and pathophysiology of lateral ankle instability. J Athl Train.

[4] Hiller CE, Kilbreath SL, Refshauge KM (2011). Chronic ankle instability: evolution of the model. J Athl Train.

[5] Wikstrom EA, Fournier KA, McKeon PO (2010). Postural control differs between those with and without chronic ankle instability. Gait Posture.

[6] Olmsted LC, Carcia CR, Hertel J, Shultz SJ (2002). Efficacy of the star excursion balance tests in detecting reach deficits in subjects with chronic ankle instability. J Athl Train.

[7] dos Santos MJ, Gorges AL, Rios JL (2014). Individuals with chronic ankle instability exhibit decreased postural sway while kicking in a single-leg stance. Gait Posture.

[8] Plisky PJ, Gorman PP, Butler RJ, Kiesel KB, Underwood FB, Elkins B (2009). The reliability of an instrumented device for measuring components of the star excursion balance test. N Am J Sports Phys Ther.

[9] Hubbard TJ, Kramer LC, Denegar CR, Hertel J (2007). Contributing factors to chronic ankle instability. Foot Ankle Int.

[10] Ross SE, Guskiewicz KM, Gross MT, Yu B (2009). Balance measures for discriminating between functionally unstable and stable ankles. Med Sci Sports Exerc.

[11] Al Adal S, Pourkazemi F, Mackey M, Hiller CE (2019). The prevalence of pain in people with chronic ankle instability: a systematic review. J Athl Train.

[12] Adal SA, Mackey M, Pourkazemi F, Hiller CE (2020). The relationship between pain and associated characteristics of chronic ankle instability: a retrospective study. J Sport Health Sci.

[13] Wikstrom EA, Song K (2019). Generic and psychological patient-reported deficits in those with chronic ankle instability: a cross sectional study. Phys Ther Sport.

[14] Charan J, Biswas T (2013). How to calculate sample size for different study designs in medical research?. Indian J Psychol Med.

[15] van Rijn RM, Willemsen SP, Verhagen AP, Koes BW, Bierma-Zeinstra SM (2011). Explanatory variables for adult patients' self-reported recovery after acute lateral ankle sprain. Phys Ther.

[16] Gribble PA, Delahunt E, Bleakley CM (2014). Selection criteria for patients with chronic ankle instability in controlled research: a position statement of the International Ankle Consortium. J Athl Train.

[17] Cao S, Wang C, Ma X (2020). Stair descent biomechanics reflect perceived instability in people with unilateral ankle sprain history. Clin Biomech (Bristol, Avon).

[18] Hiller CE, Refshauge KM, Bundy AC, Herbert RD, Kilbreath SL (2006). The Cumberland ankle instability tool: a report of validity and reliability testing. Arch Phys Med Rehabil.

[19] Hartrick CT, Kovan JP, Shapiro S (2003). The numeric rating scale for clinical pain measurement: a ratio measure?. Pain Pract.

[20] Hawker GA, Mian S, Kendzerska T, French M (2011). Measures of adult pain: Visual Analog Scale for Pain (VAS Pain), Numeric Rating Scale for Pain (NRS Pain), McGill Pain Questionnaire (MPQ), Short-Form McGill Pain Questionnaire (SF-MPQ), Chronic Pain Grade Scale (CPGS), Short Form-36 Bodily Pain Scale (SF-36 BPS), and Measure of Intermittent and Constant Osteoarthritis Pain (ICOAP). Arthritis Care Res (Hoboken).

[21] van Middelkoop M, van Rijn RM, Verhaar JA, Koes BW, Bierma-Zeinstra SM (2012). Re-sprains during the first 3 months after initial ankle sprain are related to incomplete recovery: an observational study. J Physiother.

[22] Gerber JP, Williams GN, Scoville CR, Arciero RA, Taylor DC (1998). Persistent disability associated with ankle sprains: a prospective examination of an athletic population. Foot Ankle Int.

[23] Suttmiller AMB, Cavallario JM, Baez SE, Martinez JC, McCann RS (2022). Perceived instability, pain, and psychological factors predict function and disability in individuals with chronic ankle instability. J Athl Train.

[24] Fullam K, Caulfield B, Coughlan GF, Delahunt E (2014). Kinematic analysis of selected reach directions of the star excursion balance test compared with the Y-balance test. J Sport Rehabil.

[25] Robinson RH, Gribble PA (2008). Support for a reduction in the number of trials needed for the star excursion balance test. Arch Phys Med Rehabil.

[26] Qian L, Yang X, Ma X, Yu Y, Chen WM (2022). Integration of reginal shear measurements at the foot-ground interface during routine balance assessment of the elderly population. Gait Posture.

[27] Sousa ASP, Silva A, Santos R, Sousa F, Tavares J (2013). Interlimb coordination during the stance phase of gait in subjects with stroke. Arch Phys Med Rehabil.

[28] Chen WM, Li JW, Geng X, Wang C, Chen L, Ma X (2020). The potential influence of stochastic resonance vibrations on neuromuscular strategies and center of pressure sway during single-leg stance. Clin Biomech (Bristol, Avon).

[29] Boe SG, Rice CL, Doherty TJ (2008). Estimating contraction level using root mean square amplitude in control subjects and patients with neuromuscular disorders. Arch Phys Med Rehabil.

[30] Moghadam M, Ashayeri H, Salavati M (2011). Reliability of center of pressure measures of postural stability in healthy older adults: Effects of postural task difficulty and cognitive load. Gait Posture.

[31] Freitas SMSF, Prado JM, Duarte M (2005). The use of a safety harness does not affect body sway during quiet standing. Clin Biomech.

[32] Mettler A, Chinn L, Saliba SA, McKeon PO, Hertel J (2015). Balance training and center-of-pressure location in participants with chronic ankle instability. J Athl Train.

[33] Pope M, Chinn L, Mullineaux D, McKeon PO, Drewes L, Hertel J (2011). Spatial postural control alterations with chronic ankle instability. Gait Posture.

[34] Boileau P, Zumstein M, Balg F, Penington S, Bicknell RT (2011). The unstable painful shoulder (UPS) as a cause of pain from unrecognized anteroinferior instability in the young athlete. J Shoulder Elbow Surg.

[35] Hass CJ, Bishop MD, Doidge D, Wikstrom EA (2010). Chronic ankle instability alters central organization of movement. Am J Sports Med.

[36] Moseley GL, Flor H (2012). Targeting cortical representations in the treatment of chronic pain: a review. Neurorehabil Neural Repair.

[37] Kröger S, Watkins B (2021). Muscle spindle function in healthy and diseased muscle. Skelet Muscle.

[38] Wright CJ, Arnold BL, Ross SE, Ketchum J, Ericksen J, Pidcoe P (2013). Clinical examination results in individuals with functional ankle instability and ankle-sprain copers. J Athl Train.

[39] Kwon DG, Sung KH, Chung CY (2014). Associations between MRI findings and symptoms in patients with chronic ankle sprain. J Foot Ankle Surg.

[40] Gribble PA, Hertel J, Denegar CR (2007). Chronic ankle instability and fatigue create proximal joint alterations during performance of the star excursion balance test. Int J Sports Med.

[41] Simsek S, Yagci N (2019). Acute effects of distal fibular taping technique on pain, balance and forward lunge activities in chronic ankle instability. J Back Musculoskelet Rehabil.

[42] Terada M, Harkey MS, Wells AM, Pietrosimone BG, Gribble PA (2014). The influence of ankle dorsiflexion and self-reported patient outcomes on dynamic postural control in participants with chronic ankle instability. Gait Posture.

[43] Schaefer JL, Sandrey MA (2012). Effects of a 4-week dynamic-balance-training program supplemented with Graston instrument-assisted soft-tissue mobilization for chronic ankle instability. J Sport Rehabil.

[44] Springer BA, Marin R, Cyhan T, Roberts H, Gill NW (2007). Normative values for the unipedal stance test with eyes open and closed. J Geriatr Phys Ther.

[45] Thompson CS, Hiller CE, Schabrun SM (2019). Altered spinal-level sensorimotor control related to pain and perceived instability in people with chronic ankle instability. J Sci Med Sport.

[46] El-Tallawy SN, Nalamasu R, Salem GI, LeQuang JAK, Pergolizzi JV, Christo PJ (2021). Management of musculoskeletal pain: an update with emphasis on chronic musculoskeletal pain. Pain Ther.

[47] Konradsen L, Bech L, Ehrenbjerg M, Nickelsen T (2002). Seven years follow-up after ankle inversion trauma. Scand J Med Sci Sports.

[48] Hiller CE, Nightingale EJ, Raymond J (2012). Prevalence and impact of chronic musculoskeletal ankle disorders in the community. Arch Phys Med Rehabil.

[49] Braun BL (1999). Effects of ankle sprain in a general clinic population 6 to 18 months after medical evaluation. Arch Fam Med.

[50] Luque-Suarez A, Martinez-Calderon J, Falla D (2019). Role of kinesiophobia on pain, disability and quality of life in people suffering from chronic musculoskeletal pain: a systematic review. Br J Sports Med.

[51] Suttmiller AMB, McCann RS (2021). Injury-related fear in individuals with and without chronic ankle instability: a systematic review. J Sport Rehabil.

[52] Shin HJ, Kim SH, Jeon ET, Lee MG, Lee SJ, Cho HY (2019). Effects of therapeutic exercise on sea sand on pain, fatigue, and balance in patients with chronic ankle instability: a feasibility study. J Sports Med Phys Fitness.

[53] Komenda GA, Ferkel RD (1999). Arthroscopic findings associated with the unstable ankle. Foot Ankle Int.

